# Supplementary figures and images for: Efficacy and Safety of Placental Extract on Menopausal Symptoms: A Systematic Review
Source: Nutrients. 2025 Dec 10;17(24):3857. doi: 10.3390/nu17243857 (PMC12736165; doi:10.3390/nu17243857)

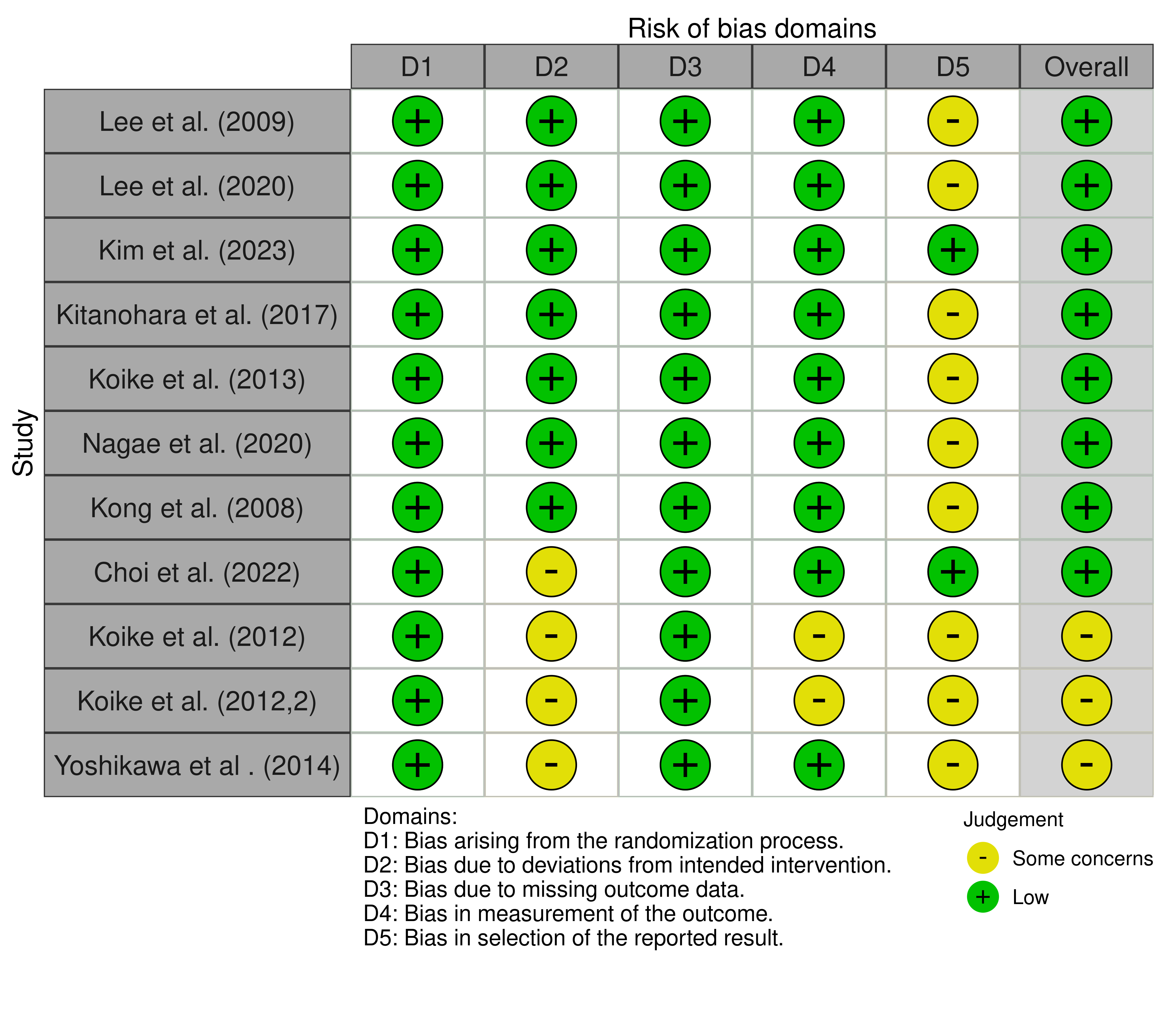

Supplement: Supplementary file 1 [file nutrients-17-03857-s001.zip › Figure S1. ROB2.png]

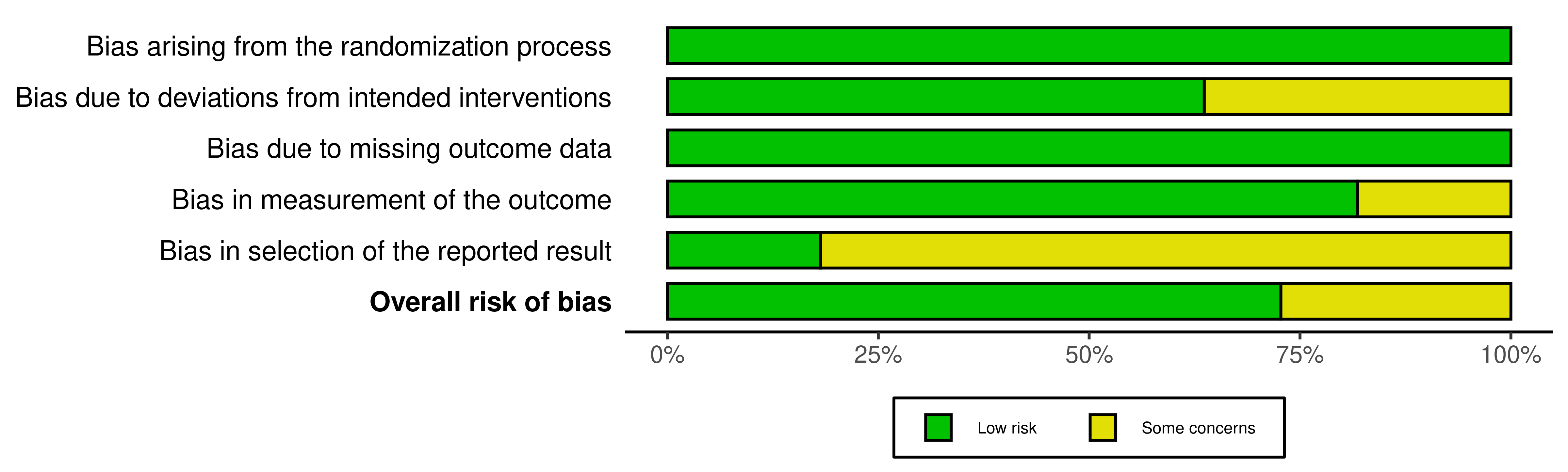

Supplement: Supplementary file 1 [file nutrients-17-03857-s001.zip › Figure S2. ROB2 (2).png]
